# Cyclosporine A loaded brain targeting nanoparticle to treat cerebral ischemia/reperfusion injury in mice

**DOI:** 10.1186/s12951-022-01474-x

**Published:** 2022-06-03

**Authors:** Daozhou Liu, Qifeng Ji, Ying Cheng, Miao Liu, Bangle Zhang, Qibing Mei, Menglei Huan, Siyuan Zhou

**Affiliations:** Department of Pharmaceutics, School of Pharmacy, Air Force Medical University, Changle West Road 169, Xi’an, 710032 Shaanxi China

**Keywords:** Cerebral ischemia/reperfusion injury, Nerve inflammation, Heavy chain ferritin, Mitochondrial permeability transition pore, Cyclosporine A, Brain targeting

## Abstract

**Background:**

Ischemic stroke is one of the main causes of death and disability in the world. The treatment for ischemic stroke is to restore blood perfusion as soon as possible. However, when ischemic brain tissue is re-perfused by blood, the mitochondrial permeability transition pore (mPTP) in neuron and microglia is excessively opened, resulting in the apoptosis of neuron and nerve inflammation. This aggravates nerve injury. Cyclosporine A (CsA) inhibits the over-opening of mPTP, subsequently reducing the release of ROS and the apoptosis of cerebral ischemia/reperfusion injured neuron and microglia. However, CsA is insoluble in water and present in high concentrations in lymphatic tissue. Herein, cerebral infarction tissue targeted nanoparticle (CsA@HFn) was developed to treat cerebral ischemia/reperfusion injury.

**Results:**

CsA@HFn efficiently penetrated the blood-brain barrier (BBB) and selectively accumulated in ischemic area, inhibiting the opening of mPTP and ROS production in neuron. This subsequently reduced the apoptosis of neuron and the damage of BBB. Consequently, CsA@HFn significantly reduced the infarct area. Moreover, CsA@HFn inhibited the recruitment of astrocytes and microglia in ischemic region and polarized microglia into M2 type microglia, which subsequently alleviated the nerve inflammation.

**Conclusions:**

CsA@HFn showed a significant therapeutic effect on cerebral ischemia/reperfusion injury by alleviating the apoptosis of neuron, nerve inflammation and the damage of BBB in ischemic area. CsA@HFn has great potential in the treatment of ischemic stroke.

**Graphical Abstract:**

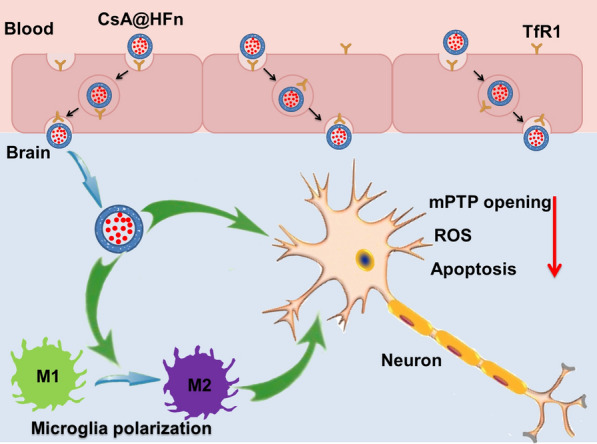

## Background

Ischemic stroke is a cerebrovascular disease with high disability and mortality rate [1, 2]. At present, the main treatment for ischemic stroke is to restore blood flow perfusion in ischemia area as soon as possible [3, 4]. However, when ischemic brain tissue is re-perfused by blood, it can cause secondary injury, which is called cerebral ischemia/reperfusion injury [5]. The mechanism of cerebral ischemia/reperfusion injury is complex, including energy metabolism disorder, oxidative stress and nerve inflammation [6–8].

Recent studies indicate that mitochondria plays an important role in cerebral ischemic injury [9, 10]. In cerebral ischemic tissue, vascular occlusion leads to insufficient supply of glucose and oxygen in local brain tissues, and neuron cells can’t produce enough adenosine triphosphate (ATP), resulting in the increase of intracellular calcium and reactive oxygen species (ROS). During reperfusion, calcium overload and oxidative stress in mitochondria of neuron lead to excessive opening of mitochondrial permeability transition pore (mPTP), which then reduces mitochondrial membrane potential and subsequently promote the release of cytochrome C. The released cytochrome C activates caspase pathway, leading to the apoptosis of neuron [11, 12]. Furthermore, the excessive opening of mPTP further leads to oxidative phosphorylation uncoupling, mitochondrial swelling and rupture of mitochondrial outer membrane. This subsequently results in the production of a large amount of reactive oxygen species (ROS) in neuron and microglia [13]. A large number of ROS further reduces the mitochondrial membrane potential and aggravates the opening of mPTP, forming a vicious cycle between the opening of mPTP and the production of ROS. This finally results in the apoptosis of neuron and the reactivity of microglia [14]. Moreover, the apoptotic neuron recruits a large number of astrocyte and microglia to the infarct area [15]. Calcium overload and oxidative stress in mitochondria of microglia promote the polarization of microglia into M1 type microglia. M1 type microglia releases a large number of pro-inflammatory factors such as IL-1β, IL-6, IFN-γ and TNF-α, which lead to nerve inflammation and exacerbate the damage of neuron. At the same time, ROS and inflammatory factors in the ischemic brain tissue activates the pre-protein of metalloproteinase, leading to the degradation of neurovascular matrix. This further deteriorates the damage of blood brain barrier (BBB) in the ischemic area [16]. Although many drugs such as antioxidant drugs, calcium antagonists, adenosine and β-blockers have been used in clinic to reduce cerebral ischemia/reperfusion injury, they have not achieved satisfactory results. There is an urgent need to develop new treatments for cerebral ischemia/reperfusion injury.

Cyclosporine A (CsA) inhibited the opening of mPTP by binding with cyclophilic protein D in mitochondria, subsequently reducing the production of ROS and the release of cytochrome C. This finally attenuated the apoptosis of neuron and reactivation microglia [17]. Moreover, studies have shown that CsA can re-educate macrophage from the pro-inflammatory M1 type to the anti-inflammatory M2 type, thereby decreasing the release of inflammatory factors [18]. Therefore, CsA has potential in the treatment of cerebral ischemia/reperfusion injury. Nevertheless, CsA is insoluble in water and widely distributed in the body [19]. In order to improve the therapeutic effect of CsA on cerebral ischemia/reperfusion injury, it is essential to construct a drug delivery system to increase solubility of CsA and deliver CsA to cerebral ischemic tissue.

Ferritin (Fn) is a naturally occurring protein in human body with a total molecular weight of about 450 kDa [20]. Fn is a spherical cage structure, which is formed by self-assembly of 24 subunits. The cage structure is usually composed of heavy chain ferritin (HFn, 21 kDa) subunits and light chain ferritin (LFn, 19 kDa) subunits. As a drug carrier, HFn does not cause toxicity and immune rejection reaction in human body [21]. In addition, HFn has unique self-assembly properties to form nanoparticle [22]. By changing pH value of solution, protein subunits of HFn undergo mutual transformation between de-polymerization and self-assembly aggregation [23]. This characteristic facilitates drug inclusion. HFn nanoparticles are hollow spheres, and it does not “hang” the drug on the surface like antibody drug conjugates, but encapsulates the drug in the lumen. Thus, HFn based drug delivery system is more stable and safe. Most importantly, as a drug vector, HFn nanoparticle can recognize and bind with transferrin receptor 1 (TfR1) on the surface of brain capillary endothelial cells [24]. Thus, HFn nanoparticles can actively penetrate BBB through TfR1 without additional modifications [25]. Moreover, HFn encapsulates drugs through depolymerizing/self-assembling, which greatly reducing the difficulty and cost of industrial production. Therefore, HFn nanoparticles are ideal CsA carrier to treat cerebral ischemia/reperfusion injury.

**Scheme 1:**
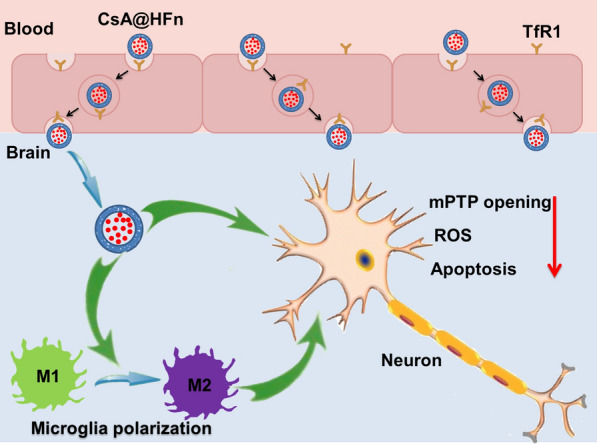
CsA@HFn improved the therapeutic effect on cerebral ischemia/reperfusion injury by inhibiting neuronal apoptosis and attenuating the inflammatory response in ischemic area

In this study, CsA loaded HFn nanoparticles (CsA@HFn) were prepared to increase the distribution of CsA in ischemic brain tissues and inhibit the over-opening of mPTP in neuron cell, subsequently alleviating the damage of neuron by decreasing the production of ROS. Besides, CsA@HFn protected neuron from damage also through reducing the area of cerebral infarction and polarizing M1 type microglia to M2 type microglia (Scheme 1).

## Materials and methods

### Materials

Cyclosporine A (CsA) was purchased from Shanghai Yuanye Biotechnology Co., Ltd (Shanghai, China). Mitochondrial membrane potential test kit (JC-1 solution) and RIPA lysate were purchased from Nanjing Beyotime Biotechnology Co. (Nanjing, China). Mitochondrial membrane permeability transition pore detection kit was purchased from Shanghai Yisheng Biotechnology Co. (Shanghai, China). β-actin antibody, CD31 antibody, CD206 antibody, CD16/32 antibody, TfR1 antibody, Iba-1 antibody and GFAP antibody were bought from Abcam (Cambridge, UK). TNF-α, IL-10 and IL-12 ELISA kits were obtained from Cloud-Clone corp (Wuhan, China). Tunel brightred apoptosis detection kit was bought from Servicebio (Wuhan, China). Cy7-NHS and FAM-NHS was obtained from Avanti Polar Lipids, Inc. (Alabaster, AL). DCFH-DA reactive oxygen assay kit and 4’,6-diamidino-2-phenylindole (DAPI) were purchased from Invitrogen Technologies Company (Carlsbad, USA). Heavy chain ferritin (HFn) was bought from Chinese Peptide Co., Ltd (Hangzhou, China). 2,3,5-triphenyltetrazolium chloride (TTC) were obtained from J&K chemical Ltd (Beijing, China). Evans blue was obtained from Sigma-Aldrich Company (Saint Louis, MI, USA). All other chemical reagents were obtained from reagent suppliers.

C57 male mice (20-25 g) were provided by the Experimental Animal Center of Air Force Medical University (Xi’an, China). Animal experiments were approved by the Air Force Medical University Institutional Animal Care and Utilization Committee (No: IACUC-20210403). SH-SY5Y cells and bEnd3 cells were purchased from CytoBiotech (Xi’an, China). SH-SY5Y cells were cultured in MEM/EBSS + DMEM/F12 medium containing 10% FBS. bEnd3 cells were cultured in DMEM medium containing 10% FBS.

### The protein expression of transferrin receptor 1 (TfR1) in oxygen-glucose deprivation/reoxygenation (OGD/R) injured SH-SY5Y cells and bEnd3 cells

SH-SY5Y cells or bEnd3 cells at logarithmic growth stage were inoculated in six-well plates (1 × 10^6^ cells/mL) and cultured for 24 h. Then the cell culture medium was replaced with low-glucose medium, and the cells were cultured for 9 h at 37 ℃ in an environment containing 94% N_2_, 5% CO_2_ and 1% O_2_. Next, the cell culture medium was replaced with DMEM complete culture medium, and the cells were cultured in a normal incubator (37 ℃, 5% CO_2_ and 95% atmosphere). 24 h later, SH-SY5Y cells or bEnd3 cells were collected. The protein expression of TfR1 in OGD/R injured SH-SY5Y cells and bEnd3 cells was detected by western blot.

### The preparation of CsA@HFn

CsA@HFn was prepared by solvent evaporation method. Briefly, HFn (50 mg) was dissolved into 50 mL acetone and water mixed solvent (v/v = 55/45). The mixture solution was sealed and stirred at room temperature for 30 min to denature the HFn. CsA (150 mg) was dissolved into 1 mL acetone, and then it was added into HFn solution. The mixture solution was sealed and stirred at room temperature for 10 min. The mixture solution was then transferred into an evaporating dish and stirred at room temperature to remove acetone. 6 h later, the mixture was filtered with 0.22 μm microporous membrane to remove the unwrapped CsA. The powder of CsA@HFn was obtained by lyophilization.

Cy7-NHS (3 mg) was dissolved into 1 mL DMSO. HFn (150 mg) was dispersed into 10 mL PBS solution. Then, Cy7-NHS solution was dropped into HFn solution. After being stirred at room temperature for 12 h, the reaction solution was transferred into the dialysis bag (molecular weight cutoff was 5000 Da) and dialyzed against water for 24 h. The water out of dialysis bag was replaced every 8 h. The solution in dialysis bag was lyophilized to obtain the solid powder of Cy7-labeled HFn (HFn-Cy7). FAM labeled HFn (HFn-FAM) was prepared as the same method as HFn-Cy7 by using FAM-NHS. Then, using HFn-Cy7 and HFn-FAM, Cy7 labeled CsA@HFn (CsA@HFn-Cy7) and FAM labeled CsA@HFn (CsA@HFn-FAM) were prepared by using the same method in the preparation of CsA@HFn.

### Characterization of CsA@HFn

The particle size and zeta potential of CsA@HFn were measured by zeta potential and laser particle size analyzer (Delsa Nano C, Beckman, USA). Appropriate amount of CsA@HFn solution was dropped onto the screen covered with supporting film. After natural drying, CsA@HFn was stained with phosphotungstic acid. The particle size and morphology of CsA@HFn were observed under transmission electron microscope (TEM, Hitachi, Japan). Appropriate amount of CsA@HFn was dispersed in distilled water, PBS (pH 7.4), 10% FBS and DMEM solutions, respectively. The stability of CsA@HFn was investigated by using zeta potential and laser particle size analyzer.

The drug loading of CsA@HFn was determined by high performance liquid chromatography (HPLC, Waters 2695/2996, USA). The determination was performed on Dikma ODS C_18_ column (250 mm×4.6 mm, 5 μm), and detection wavelength was set at 210 nm. The mobile phase composition was acetonitrile/water = 90/10 (V/V) at the flow rate of 1.0 mL/min. The column temperature was 55 ℃. 10 mg of CsA@HFn freeze-dried powder was dissolved into 10 mL acetone. After filtration with 0.22 μm microporous membrane, 20 µL of filtrate was injected into HPLC system to detect CsA loading in CsA@HFn according to the above chromatographic conditions. The drug loading was calculated as following equation: drug loading = [the weight of CsA in CsA@HFn/weight of CsA@HFn]×100%.

In vitro drug release of CsA@HFn was investigated by HPLC. Appropriate amount of CsA@HFn was dispersed into 5 mL PBS solutions with different pH (5.0, 6.8 and 7.4) or 5 mL sodium hypochlorite solution at different concentration (0.1 and 10 µM). Then the dispersion was transferred into a dialysis bag (molecular weight cutoff was 5000 Da) and immerged into 60 mL PBS solution or sodium hypochlorite solution with the same as that in dialysis bag. At 1, 2, 4, 8, 12, 24, 48 and 96 h, 200 µL of dispersion was taken out from dialysis bag, and the concentration of CsA was determined by HPLC. The cumulative drug release was calculated, and the drug release curve was plotted.

### The uptake of CsA@HFn by OGD/R injured SH-SY5Y cells

SH-SY5Y cells at logarithmic growth stage were inoculated in six-well plates (1 × 10^6^ cells/mL) and cultured for 24 h. Then the cell culture medium was replaced with low-glucose medium, and the cells were cultured for 9 h in an environment containing 94% N_2_, 5% CO_2_ and 1% O_2_. Next, the cell culture medium was replaced with DMEM medium containing CsA@BSA-FAM and CsA@HFn-FAM, and the cells were cultured in a normal incubator (37 ℃, 5% CO_2_ and 95% atmosphere) for 1, 2 and 4 h. Cells were collected and washed for 3 times with PBS buffer. Finally, cells were dispersed in 400 µL PBS, and the uptake of CsA@HFn in OGD/R injured SH-SY5Y cells was investigated using flow cytometer (BD0617, USA).

To investigate the uptake mechanism of CsA@HFn by OGD/R injured SH-SY5Y cells, SH-SY5Y cells at logarithmic growth stage were inoculated in six-well plates (1 × 10^6^ cells/mL) and cultured for 24 h. Then the cell culture medium was replaced with low-glucose medium, and the cells were cultured for 9 h in an environment containing 94% N_2_, 5% CO_2_ and 1% O_2_. Next, the cell culture medium was replaced with DMEM medium containing chlorpromazine solution (10 µg/mL), colchicine solution (800 µg/mL), methyl-β-cyclodextrin solution (5 µg/mL), transferrin (Tf, 100 µg/mL) and 2-deoxy-d-glucose (900 µg/mL), and cells were cultured in a normal incubator (37 ℃, 5% CO_2_ and 95% atmosphere) for 1 h. After that, cell culture medium containing CsA@BSA-FAM and CsA@HFn-FAM was added, and the cells were cultured for another 4 h. Cells were collected and washed for 3 times with PBS. Finally, cells were dispersed in 400 µL PBS, and the uptake of CsA@HFn by OGD/R injured SH-SY5Y cells was investigated using flow cytometer.

### Efficiency of CsA@HFn penetrating in vitro BBB

Bend3 cells (1 × 10^5^ cells/mL, 300 µL) at logarithmic growth stage were inoculated into donor chamber of 24-well transwell, and DMEM medium containing 10% FBS was added into recipient chamber of 24-well transwell. The culture medium was changed every 2 days. On 7th day, the resistance value between the recipient and donor chamber was measured by an electrical resistance meter. When the resistance value exceeded 200 Ω/cm^2^, the in vitro BBB model was considered to be successfully set up [26]. The culture medium in the donor chamber and recipient chamber were removed. Fresh CsA@HFn containing serum-free DMEM medium was added into donor chamber (CsA concentration was 50 µg/mL), and fresh serum-free DMEM medium was add into recipient chamber. After cells were incubated for 1, 3, 6 and 12 h, the culture medium in recipient chamber was collect and lyophilized. The solid powder was dissolved into 200 µL acetone, and the solution was filtrated with 0.22 μm microporous membrane. The concentration of CsA in filtrate was detected by HPLC, and the transport efficiency of CsA@HFn across in vitro BBB was calculated. In addition, transferrin (Tf, 100 µg/mL) was added into donor chamber. 2 h later, CsA@HFn was added into donor chamber, and cells were incubated for 1, 3, 6 and 12 h. The effect of Tf on CsA@HFn penetrating in vitro BBB was also investigated *via* HPLC.

### Effects of CsA@HFn on the apoptosis of SH-SY5Y cells induced by OGD/R

SH-SY5Y cells at logarithmic growth stage were inoculated in six-well plates (1 × 10^6^ cells/mL) and cultured for 24 h. Then the cell culture medium was replaced with low-glucose medium, and the cells were cultured for 9 h in an environment containing 94% N_2_, 5% CO_2_ and 1% O_2_. Next, the cell culture medium was replaced with DMEM medium containing CsA, CsA@BSA and CsA@HFn, and the cells were cultured in a normal incubator (37 ℃, 5% CO_2_ and 95% atmosphere) for 24 h. SH-SY5Y cells were collected and stained with Annexin V/PI. After that, cells were dispersed in 400 µL PBS, and the apoptosis of SH-SY5Y cells was detected by flow cytometer.

### Effect of CsA@HFn on mPTP in OGD/R injured SH-SY5Y cells

mPTP assay kit was used to detect the effect of CsA@HFn on the over-opening of mPTP in OGD/R injured SH-SY5Y cells. The fluorescent probe calcein acetylmethyl ester (Calcein AM) is passively transported into living cells and catalyzed by esterase to produce calcein, which make SH-SY5Y cells show strong green fluorescence. After SH-SY5Y cells are treated with cobalt chloride, calcein is combined with cobalt ions to form non-fluorescent calcein-2Co. Under normal circumstances, the opening of mPTP is low and cobalt ions can’t enter mitochondria. Thus, only mitochondria present green fluorescence at this time. When SH-SY5Y cells are injured by OGD/R, mPTP is over-opened, and calcein is released from mitochondria. The released calcein is combined with cobalt ions in cytoplasm and subsequently lose its fluorescence. At the same time, cobalt ions can also enter mitochondria to form non-fluorescent calcein-2Co, resulting in the weakening of green fluorescence in SH-SY5Y cells. In this way, the opening degree of mPTP can be judged according to the intensity of calcein green fluorescence in SH-SY5Y cells. The stronger the green fluorescence becomes, the lower the opening of mPTP is [27].

SH-SY5Y cells at logarithmic growth stage were inoculated in six-well plates (1 × 10^6^ cells/mL) and cultured for 24 h. Then the cell culture medium was replaced with low-glucose medium, and the cells were cultured for 9 h in an environment containing 94% N_2_, 5% CO_2_ and 1% O_2_. Next, the cell culture medium was replaced with DMEM medium containing CsA, CsA@BSA and CsA@HFn, and the cells were cultured in a normal incubator (37 ℃, 5% CO_2_ and 95% atmosphere) for 24 h. After that, the cell culture medium was replaced with DMEM medium containing Calcein-AM, and the cells were cultured for 20 min. Cells were washes with PBS for 3 times. Then, the cell culture medium was replaced with DMEM medium containing cobalt chloride, and the cells were cultured for 30 min. Cells were washed with PBS for 3 times. Finally, SH-SY5Y cells were collected and dispersed in PBS. The fluorescence intensity in SH-SY5Y cells was measured by fluorescence spectrophotometer (Hitachi, F-2700, Japan).

### Effects of CsA@HFn on mitochondrial membrane potential in OGD/R injured SH-SY5Y cells

Mitochondrial membrane potential detection kit was used to detect the effect of CsA@HFn on mitochondrial membrane potential in OGD/R injured SH-SY5Y cells. JC-1 can aggregate in the mitochondrial matrix with high membrane potential, showing red fluorescence. However, when mitochondria are damaged, the mitochondrial membrane potential is decreased. JC-1 can’t aggregate, showing green fluorescence. Therefore, the ratio between red fluorescence and green fluorescence reflects the mitochondrial membrane potential. The higher ratio of red/green fluorescence intensity becomes, the higher mitochondrial membrane potential is [27].

SH-SY5Y cells at logarithmic growth stage were inoculated in six-well plates (1 × 10^6^ cells/mL) and cultured for 24 h. Then the cell culture medium was replaced with low-glucose medium, and the cells were cultured for 9 h in an environment containing 94% N_2_, 5% CO_2_ and 1% O_2_. Next, the cell culture medium was replaced with DMEM medium containing CsA, CsA@BSA and CsA@HFn, and the cells were cultured in a normal incubator (37 ℃, 5% CO_2_ and 95% atmosphere) for 24 h. After that, the cell culture medium was replaced with DMEM medium containing JC-1, and the cells were cultured for 30 min. Cells were washed with PBS for 3 times. Finally, SH-SY5Y cells were collected and dispersed in PBS. The red and green fluorescence intensity in SH-SY5Y cells was measured by fluorescence spectrophotometer.

### Effects of CsA@HFn on reactive oxygen species in OGD/R injured SH-SY5Y cells

Reactive oxygen species (ROS) in SH-SY5Y cells were detected by fluorescence probe DCFH-DA. Non-fluorescent DCFH-DA can be hydrolyzed by intracellular esterase to produce DCFH. DCFH can’t penetrate the cell membrane, and it can be oxidized by intracellular ROS to produce fluorescent DCF. The ROS content in cells is proportional to fluorescence intensity of DCF [28, 29].

SH-SY5Y cells at logarithmic growth stage were inoculated in six-well plates (1 × 10^6^ cells/mL) and cultured for 24 h. Then the cell culture medium was replaced with low-glucose medium, and the cells were cultured for 9 h in an environment containing 94% N_2_, 5% CO_2_ and 1% O_2_. Next, the cell culture medium was replaced with DMEM medium containing CsA, CsA@BSA and CsA@HFn, and the cells were cultured in a normal incubator (37 ℃, 5% CO_2_ and 95% atmosphere) for 24 h. After that, the cell culture medium was replaced with DMEM medium containing DCFH-DA (10 µM), and the cells were cultured for 20 min. Cells were washes with PBS for 3 times. Finally, SH-SY5Y cells were collected and dispersed in PBS. The fluorescence in SH-SY5Y cells was observed by fluorescence microscope.

### Cerebral ischemic/reperfusion injury model in mice

The middle cerebral artery occlusion (MCAO) model in mice was established by suture-occluded method [30]. In short, mice were anesthetized with isoflurane and held on a thermostat. Then, the filament with silicone tip was inserted into the external carotid artery of mice. 1 h after hypoxia, the filament was pulled out, and incision was sutured.

### Distribution of CsA@HFn in MCAO mice

After suture plug was pulled out, CsA@HFn-Cy7 (equivalent CsA dose was 2.5 mg/kg) was immediately injected into the tail vein of the MCAO mice. 24 h later, the brain tissue was obtained, and TTC staining was performed. The distribution of CsA@HFn-Cy7.5 in the brain was observed by Caliper IVIS Lumina II (Siemens, Germany).

After suture plug was pulled out, CsA@HFn-FAM (equivalent CsA dose was 2.5 mg/kg) was immediately injected into the tail vein of the MCAO mice. 24 h later, the brain tissue was collected and fixed with 4% paraformaldehyde. Paraffin sections of brain tissue were prepared. The nucleus was stained with DAPI. The blood vessel was labeled with CD31 antibody, and the distribution of CsA@HFn-FAM in cerebral infarction area and normal brain tissue was observed by laser scanning confocal microscopy (LSCM, Olympus, Japan).

### Therapeutic effect of CsA@HFn on cerebral ischemia/reperfusion injury in mice

After suture plug was pulled out, normal saline, CsA (2.5 mg/kg), CsA@BSA (equivalent CsA dose was 2.5 mg/kg) and CsA@HFn ((equivalent CsA dose was 1.25 and 2.5 mg/kg) was immediately injected into the tail vein of the MCAO mice. The sham group was used as control. 24 h after drug administration, the neurological function of each group was scored by 5-point method [31].

24 h after drug preparations were injected into the tail vein of MCAO mice, the intact brain tissues were obtained and cut into 5 slices. The sections were stained with TTC, and the effects of CsA@HFn on cerebral infarction size in MACO mice were observed by stereomicroscopy. Paraffin-embedded brain tissue was sectioned, and then *H&E* staining, tunel staining, nissl staining and silver plating staining were performed to investigate the protective effect of CsA@HFn on neuron in cerebral infarction area of MACO mice.

24 h after drug preparations were injected into the tail vein of MCAO mice, the intact brain tissue was collected, and frozen sections were prepared. DCFH-DA reactive oxygen assay kit was used to observe the effect of CsA@HFn on ROS in cerebral infarction region.

24 h after drug preparations were injected into the tail vein of MCAO mice, evens blue solution was injected *via* tail vein. 30 min later, MCAO mice was perfused with normal saline through heart. Then intact brain tissue was obtained. The distribution of evens blue in brain tissue was observed to investigate the integrity of BBB.

### Effects of CsA@HFn on inflammatory microenvironment in cerebral ischemia region in MCAO mice

24 h after drug preparations were injected into the tail vein of MCAO mice, the intact brain tissues were obtained. Then, paraffin-embedded brain tissues were prepared and sectioned. The protein expression of glial fibrillary acidic protein (GFAP, marker of astrocyte) and ionized calcium binding adaptor molecule 1 (Iba-1, marker of microglia) in MCAO mice brain sections were detected by IHC staining. M1 type and M2 type microglia were labeled by CD16/32 and CD206 respectively to observe the proportion of M1 type and M2 type microglia in cerebral infarction area [32]. The M1 type microglia-associated factor such as interleukin-12 (IL-12), tumor necrosis factor-α (TNF-α) and M2 type microglia-associated factor interleukin-10 (IL-10) in infarcted area was detected by ELISA.

### In vivo safety evaluation of CsA@HFn

24 h after drug preparations were injected into the tail vein of MCAO mice, blood was taken via orbital. Then blood was centrifuged and serum was collected. The activities of alanine transaminase (ALT) and aspartate aminotransferase (AST) in supernatant, as well as the contents of serum creatinine and serum urea nitrogen were detected by automatic biochemical analyzer. The brain, heart, liver, spleen, lung and kidney of MCAO mice in each group were obtained and fixed with 4% paraformaldehyde for 24 h. Paraffin-embedded tissue was sectioned, and *H&E* staining was performed. The effect of CsA@HFn on normal tissue morphology was observed by inverted microscope.

### Statistical analysis

All data were expressed as mean ± SD, and SPSS 18.0 software was used for one-way ANOVA.

## Results

### Characterization of CsA@HFn

**Fig. 1:**
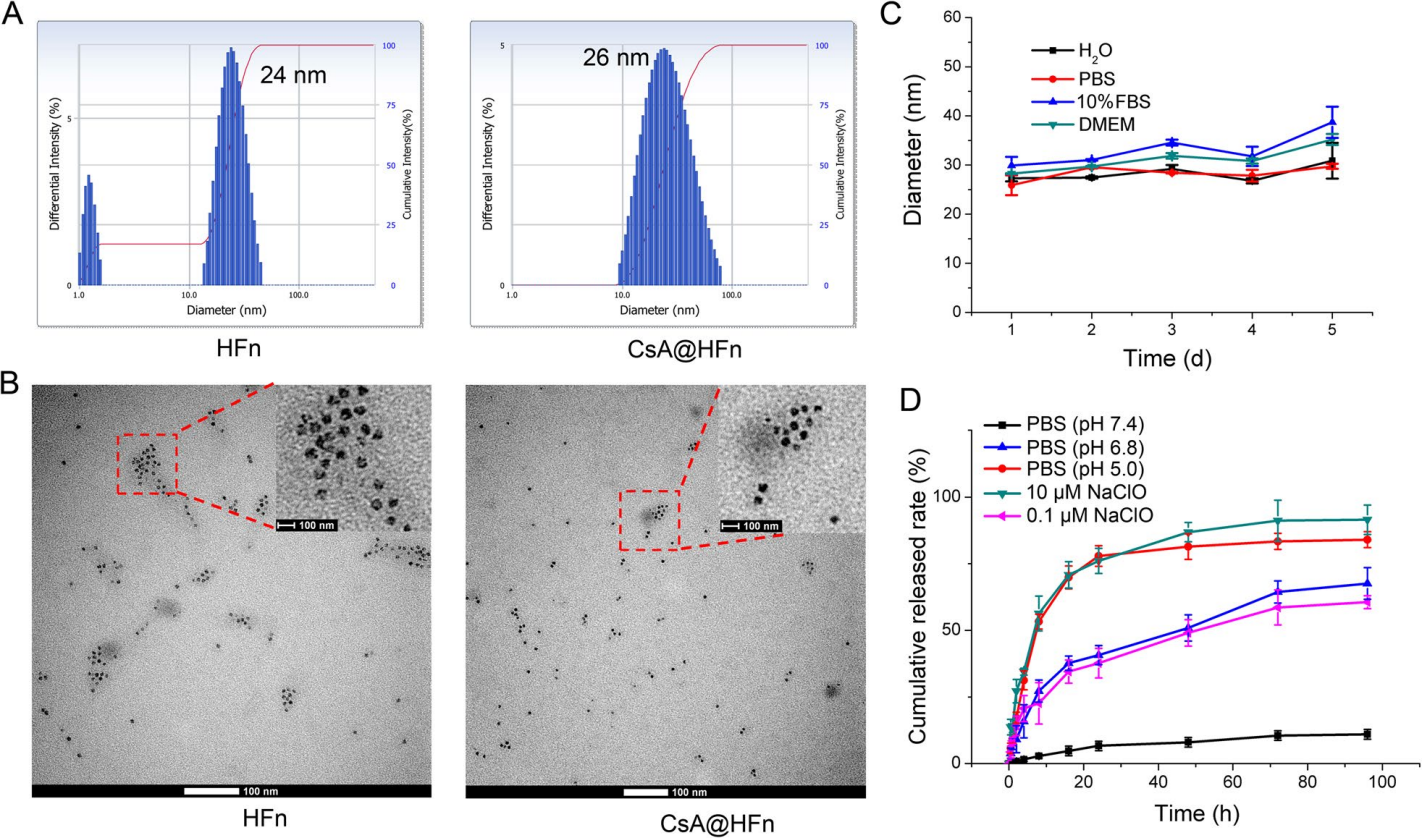
Characterization of CsA@HFn. **A** The particle size distribution of HFn and CsA@HFn. **B** The TEM image of HFn and CsA@HFn. **C** The stability of CsA@HFn in distilled water, PBS, 10% FBS solution and DMEM solution. **D** The cumulative CsA release from CsA@HFn in PBS at different pH value and PBS containing NaClO (pH7.4) solution. (n = 3, $\stackrel{-}{x}$ ± SD)

The particle size of CsA@HFn was 26 nm, which is basically similar to that of HFn (Fig. 1A). The drug loading of CsA in CsA@HFn was 10.4%. TEM result showed that HFn nanoparticles had a hollow structure. But after CsA was encapsulated in HFn nanoparticles, CsA@HFn displayed a uniform structure (Fig. 1B), proving that CsA was encapsulated in HFn nanoparticles. When CsA@HFn was dispersed in distilled water, PBS buffer, 10% FBS solution and DMEM solution, the particle size of CsA@HFn did not change significantly within 5 days, suggesting that CsA@HFn had good stability (Fig. 1 C). The cumulative release rate of CsA from CsA@HFn significantly increased in pH 6.8 PBS and pH 5.0 PBS. More than 40% and 77% of CsA was released from CsA@HFn within 24 h in pH 6.8 PBS and pH 5.0 PBS, respectively. Besides, the cumulative release rate increased with the increase of sodium hypochlorite concentration (Fig. 1D). This indicated that CsA@HFn exhibited pH-sensitive and ROS-sensitive drug release characteristics.

### Protective effect of CsA@HFn on OGD/R injured SH-SY5Y cells

**Fig. 2:**
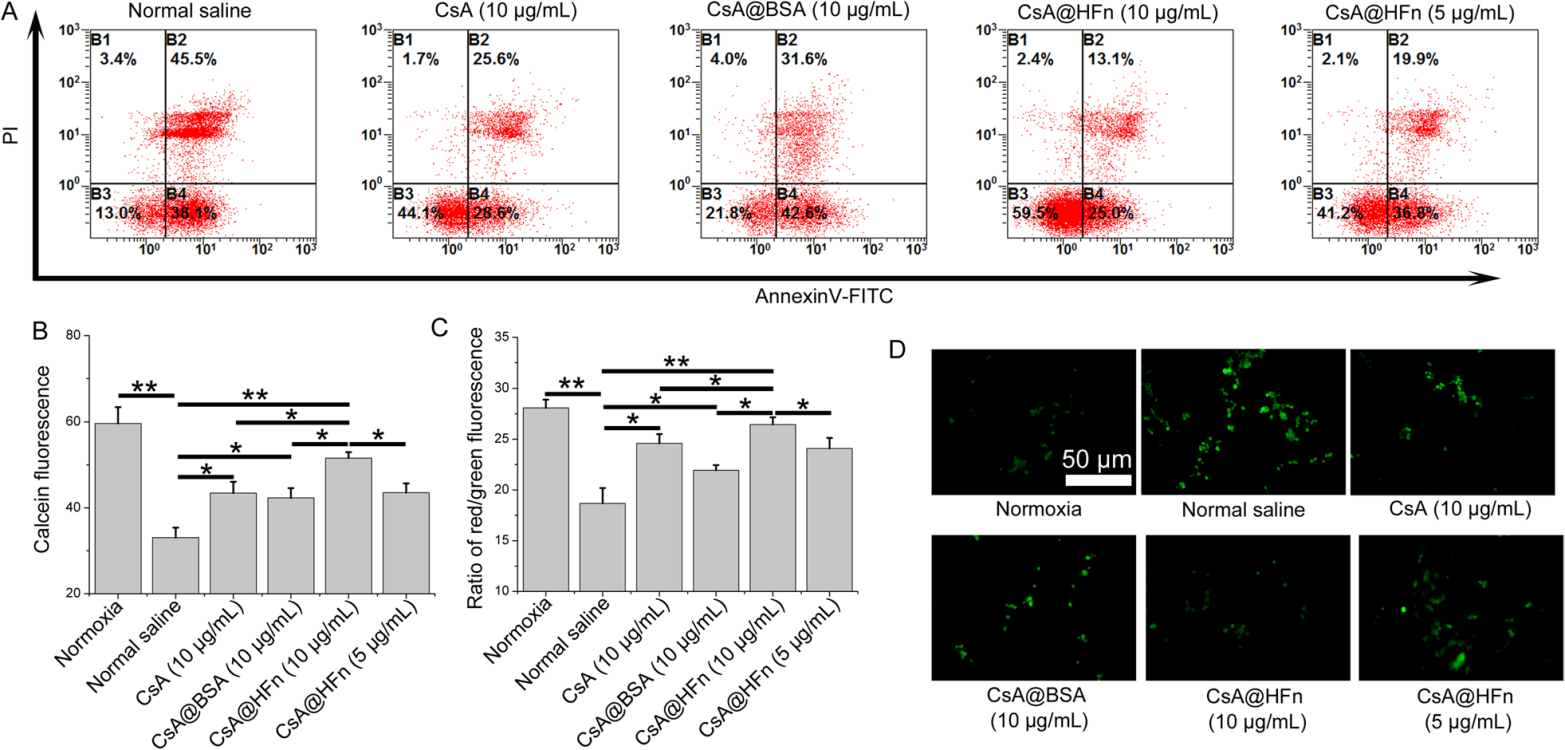
Protective effect of CsA@HFn on OGD/R injured SH-SY5Y cells. **A** Effects of CsA@HFn on the apoptosis of OGD/R injured SH-SY5Y cells. **B** Effects of CsA@HFn on the opening of mPTP in OGD/R injured SH-SY5Y cells. **C** Effects of CsA@HFn on the mitochondrial membrane potential in OGD/R injured SH-SY5Y cells. D Effects of CsA@HFn on ROS level in OGD/R injured SH-SY5Y cells. (n = 3, x ®± SD, *P < 0.05; **P < 0.01)

Firstly, the effect of CsA@HFn on the apoptosis of OGD/R injured SH-SY5Y cells was investigated by flow cytometer. The results showed that a large number of SH-SY5Y cells were apoptotic in the control group, the proportion of early apoptotic cells and late apoptotic cells was 38.1% and 45.5%, respectively. CsA@HFn inhibited the apoptosis of SH-SY5Y cells in concentration-dependent manner. As compared with CsA and CsA@BSA, CsA@HFn treatment group exhibited the least apoptotic SH-SY5Y cells (Fig. 2A).

Secondly, the effect of CsA@HFn on the opening of mPTP in OGD/R injured SH-SY5Y cells was detected by mPTP assay kit. The results showed that the fluorescence intensity in normoxia group was the highest, while the fluorescence intensity in OGD/R injury group was the lowest, indicating that OGD/R led to the over-opening of mPTP in SH-SY5Y cells. CsA@HFn, CsA and CsA@BSA increased the fluorescence intensity in SH-SY5Y cells, and CsA@HFn treated SH-SY5Y cells displayed the highest fluorescence intensity, indicating that CsA@HFn could significantly inhibit the over-opening of mPTP in OGD/R injured SH-SY5Y cells (Fig. 2B).

Thirdly, the effect of CsA@HFn on mitochondrial membrane potential of OGD/R injured SH-SY5Y cells was detected by using mitochondrial membrane potential detection kit. The results showed that compared with the normoxia group, the ratio between red and green fluorescence intensity in OGD/R injury group was significantly reduced, indicating that OGD/R caused the damage of mitochondria in SH-SY5Y cells. CsA@HFn, CsA and CsA@BSA improved the ratio between red and green fluorescence intensity in OGD/R injured SH-SY5Y cells, and the ratio between red and green fluorescence intensity was the highest in CsA@HFn treated group, indicating that CsA@HFn significantly recovered mitochondrial membrane potential in OGD/R injured SH-SY5Y cells (Fig. 2C).

Finally, DCFH-DA probe was used to investigate the effect of CsA@HFn on ROS content in OGD/R injured SH-SY5Y cells. The results showed that ROS content in OGD/R injured SH-SY5Y cells was significantly increased as compared with that in normoxia group. CsA@HFn, CsA and CsA@BSA reduced ROS content in OGD/R injured SH-SY5Y cells. In addition, CsA@HFn significantly reduced ROS level in OGD/R injured SH-SY5Y cells than CsA and CsA@BSA did (Fig. 2D).

### The uptake of CsA@HFn in OGD/R injured SH-SY5Y cells

**Fig. 3:**
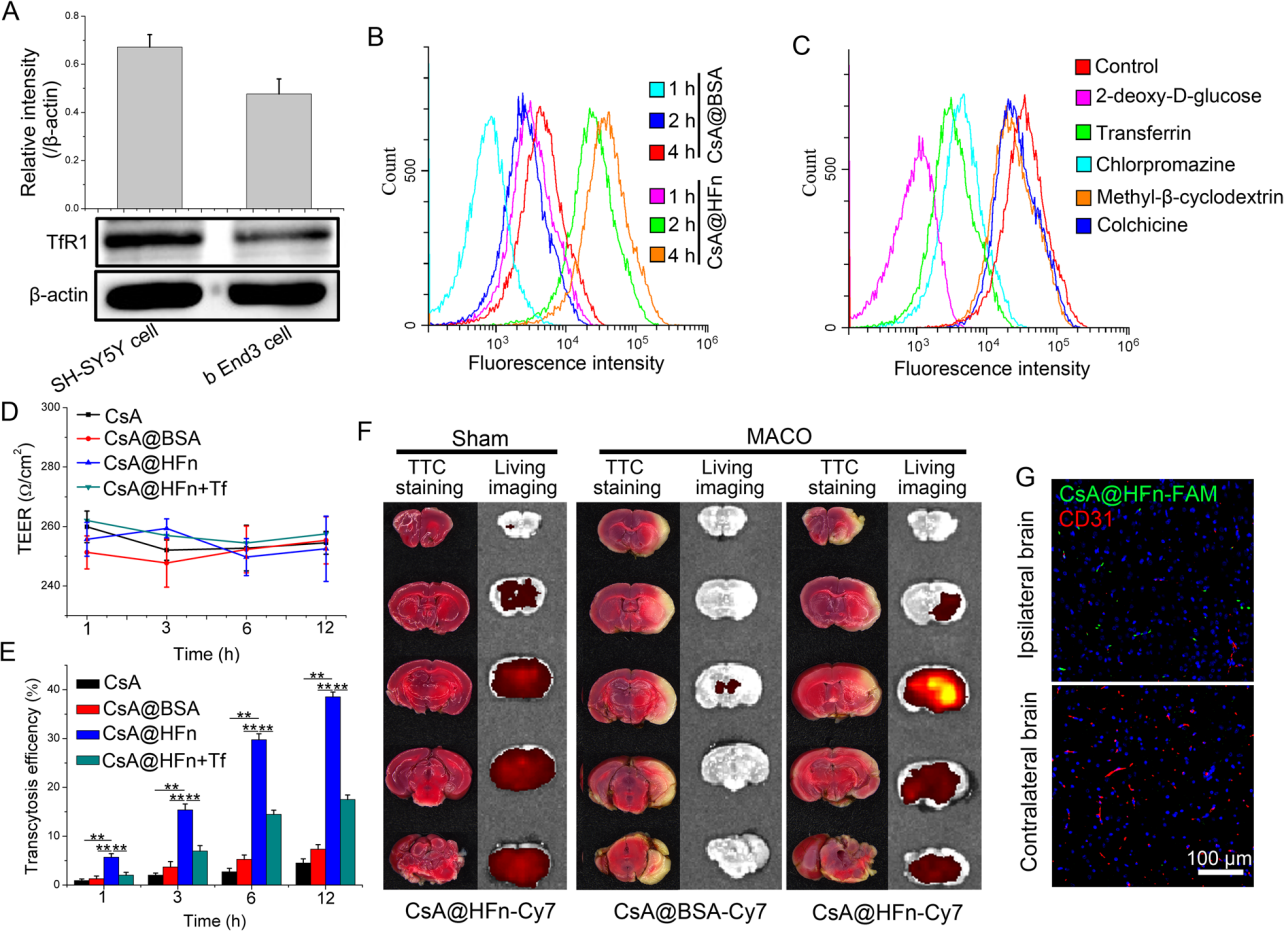
The uptake of CsA@HFn in OGD/R injured SH-SY5Y cells and penetration BBB efficiency of CsA@HFn. **A** The protein expression of TfR1 in OGD/R injured SH-SY5Y cells and bEnd3 cells. **B** The uptake of CsA@HFn in OGD/R injured SH-SY5Y cells. **C** The uptake mechanism of CsA@HFn in OGD/R injured SH-SY5Y cells. **D** The resistance values between transwell recipient chamber and donor chamber within 12 h after drug administration. **E** The penetration in vitro BBB efficiency of CsA@HFn. **F** The distribution of CsA@HFn in brain tissue of MCAO mice. **G** The distribution of CsA@HFn in infarcted area and normal area of MCAO mice (green color stands for nanoparticle, and red color stands for blood vessel). (n = 3, x ®±SD, **P < 0.01)

Firstly, western blot was used to investigate the protein expression of TfR1 in OGD/R injured SH-SY5Y cells and bEnd3 cells. The results showed that the TfR1 was highly expressed in OGD/R injured SH-SY5Y cells and bEnd3 cells (Fig. 3A).

Secondly, the uptake of CsA@HFn by OGD/R injured SH-SY5Y cells was detected by flow cytometer. The results showed that CsA@HFn was taken up by OGD/R injured SH-SY5Y cells in a time-dependent manner, and the accumulation of CsA@HFn in OGD/R injured SH-SY5Y cells was significantly higher than that of CsA@BSA (Fig. 3B).

Finally, the uptake mechanism of CsA@HFn in OGD/R injured SH-SY5Y cells was investigated by flow cytometer. The results showed that the uptake of CsA@HFn by OGD/R injured SH-SY5Y cells was significantly reduced after pretreatment cells with sodium azide, 2-deoxyglucose and 4 ℃, indicating that the uptake of CsA@HFn by OGD/R injured SH-SY5Y cells was an active process, which depended on energy and carrier. After pretreatment cells with chlorpromazine, the uptake of CsA@HFn by OGD/R injured SH-SY5Y cells was significantly decreased, indicating that uptake of CsA@HFn by OGD/R injured SH-SY5Y cell was mainly through clathrin-mediated endocytosis. Moreover, the uptake of CsA@HFn by OGD/R injured SH-SY5Y cells was significantly reduced in the present of transferrin (Tf), suggesting that TfR1 mediated the cellular uptake of CsA@HFn in OGD/R injured SH-SY5Y cells (Fig. 3C).

### Transportation of CsA@HFn across in vitro BBB

In order to investigate the ability of CsA@HFn penetrating BBB, an in vitro BBB model was set up. Within 12 h after drug was added, there was no significant difference in resistance values between recipient chamber and donor chamber of transwell, indicating that drug treatment did not change the integrity of in vitro BBB (Fig. 3D). As shown in Fig. 3E, CsA@HFn could penetrate in vitro BBB in a time-dependent manner, and its transport efficiency was significantly higher than that of free CsA and CsA@BSA. When bEnd3 cells in the donor chamber was pretreated with transferrin, the transport efficiency of CsA@HFn across in vitro BBB was significantly decreased, indicating that TfR1 mediated the transcytosis of CsA@HFn.

### Biodistribution of CsA@HFn in MCAO mice

As shown in Fig. 3F, after TTC staining, the brain tissue of sham mice showed red color, while the brain tissue of MCAO mice in the infarcted area showed white color, proving that the MCAO model was successfully set up. In sham group, the distribution of CsA@HFn-Cy7 in the brain tissue was less. But in MCAO group, the distribution of CsA@HFn-Cy7 in the brain tissue was significantly increased. Moreover, the distribution of CsA@HFn-Cy7 in cerebral infarction area was significantly higher than the in normal brain tissue. As compared with CsA@HFn-Cy7, the distribution of CsA@BSA-Cy7 in brain tissue of MCAO mice was significantly reduced. After using FAM to label CsA@HFn and using CD31-Cy3 antibody to label the blood vessels, the distribution of CsA@HFn-FAM in the cerebral infarction area was observed via LSCM. The results showed that the green fluorescence of CsA@HFn-FAM was separated from the red fluorescence of CD31 (Fig. 3G), indicating that the CsA@HFn could penetrate blood vessels and diffuse into the tissue of the cerebral infarction area. In normal brain tissue, there was almost no distribution of CsA@HFn-FAM. These results indicated that CsA@HFn-FAM could be selectively delivered to cerebral infarction area.

### The effect of CsA@HFn on infarct area and neurological function in MCAO mice

**Fig. 4:**
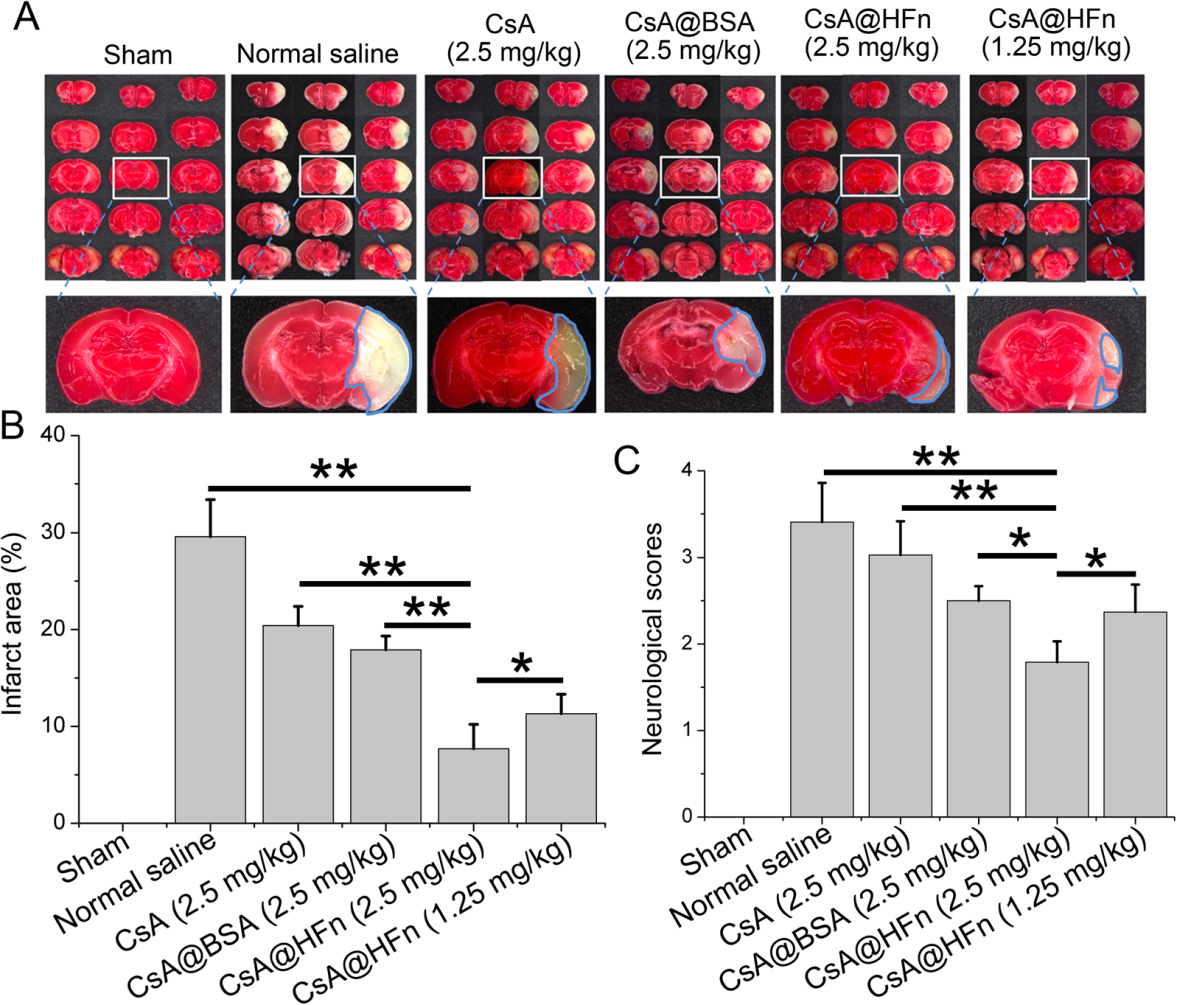
The effect of CsA@HFn on infarct area and neurological function in MCAO mice. **A** TTC staining of brain tissue sections. White color stands for infarcted areas; red color stands for non-infarcted areas. **B** Statistical analysis of infarct area. **C** Neurological scores of MCAO mice. (n = 3, x ®± SD, *P < 0.05; **P < 0.01)

TTC staining of brain tissue sections showed that CsA@HFn dose-dependently reduced the infarct area in MCAO mice, and infarct area in CsA@HFn treated MCAO mice was less than that in CsA@BSA and free CsA treated MCAO mice (Fig. 4A, B). In order to investigate the neuroprotective effect of CsA@HFn, the neurological function of mice was scored by 5-point method. The results showed that MCAO mice displayed the highest neurological function score, and CsA@HFn treatment MCAO mice displayed the lowest neurological function score, suggesting that CsA@HFn alleviated the damage of the nerve system caused by cerebral ischemia-reperfusion (Fig. 4C).

### Protective effect of CsA@HFn on neuron in MCAO mice

**Fig. 5:**
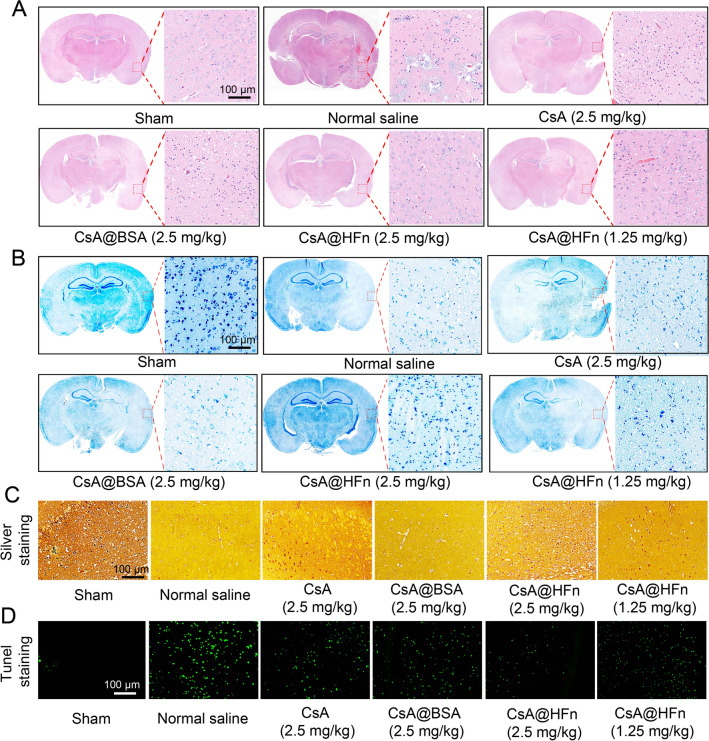
Protective effect of CsA@HFn on neuron in MCAO mice. **A** *H&E* staining of brain tissue in MCAO mice. **B** Nissl staining of brain tissue in MCAO mice. **C** Silver staining of brain tissue in MCAO mice. **D** Tunel staining of brain tissue in MCAO mice. (n = 3)

H&E staining of brain tissue in MCAO mice is showing in Fig. 5A. When MCAO mice were treated with normal saline, large necrosis occurred in cerebral infarction tissue. There were neuronal chromatin agglutination, nuclear pyknosis and numerous inflammatory cells in cerebral infarction tissue. When MCAO mice were treated with free CsA and CsA@BSA, the necrosis in cerebral infarction tissue was reduced, but the nuclear pyknosis of neuron still existed in large numbers. After MCAO mice were treated with CsA@HFn, the necrosis in cerebral infarction tissue was significantly reduced, and the chromatin of neuron was in the shape of three lobes, which showed no significant difference from normal neuron. Nissl staining of cerebral infarction tissue showed that as compared with normal saline, free CsA and CsA@BSA, CsA@HFn increased the density of neuron in cerebral infarction tissue in a dose-dependent manner (Fig. 5B). The results of silver staining showed that as compared with the sham group, the silver staining in cerebral infarction region of MCAO mice was significantly reduced, indicating that cerebral ischemia reperfusion caused nerve myelin injury. CsA, CsA@BSA and CsA@HFn increased the silver staining in cerebral infarction region in MCAO mice. As compared with CsA and CsA@BSA, CsA@HFn markedly increased silver staining in cerebral infarction region in treated MCAO mice, suggesting that CsA@HFn reduced nerve myelin injury (Fig. 5C). Tunel staining in cerebral infarction tissue in MCAO mice showed that there were a large number of tunel positive cells in cerebral infarction tissue in normal saline treated MCAO mice, indicating that there was a large number of apoptotic neuron in cerebral infarction tissue in MCAO mice. CsA@HFn dose-dependently reduced the apoptotic neuron in cerebral infarction tissue. As compared with CsA and CsA@BSA, CsA@HFn significantly decreased the number of apoptotic neuron in cerebral infarction tissue (Fig. 5D).

### Effects of CsA@HFn on inflammatory microenvironment in cerebral infarction area in MCAO mice

**Fig. 6:**
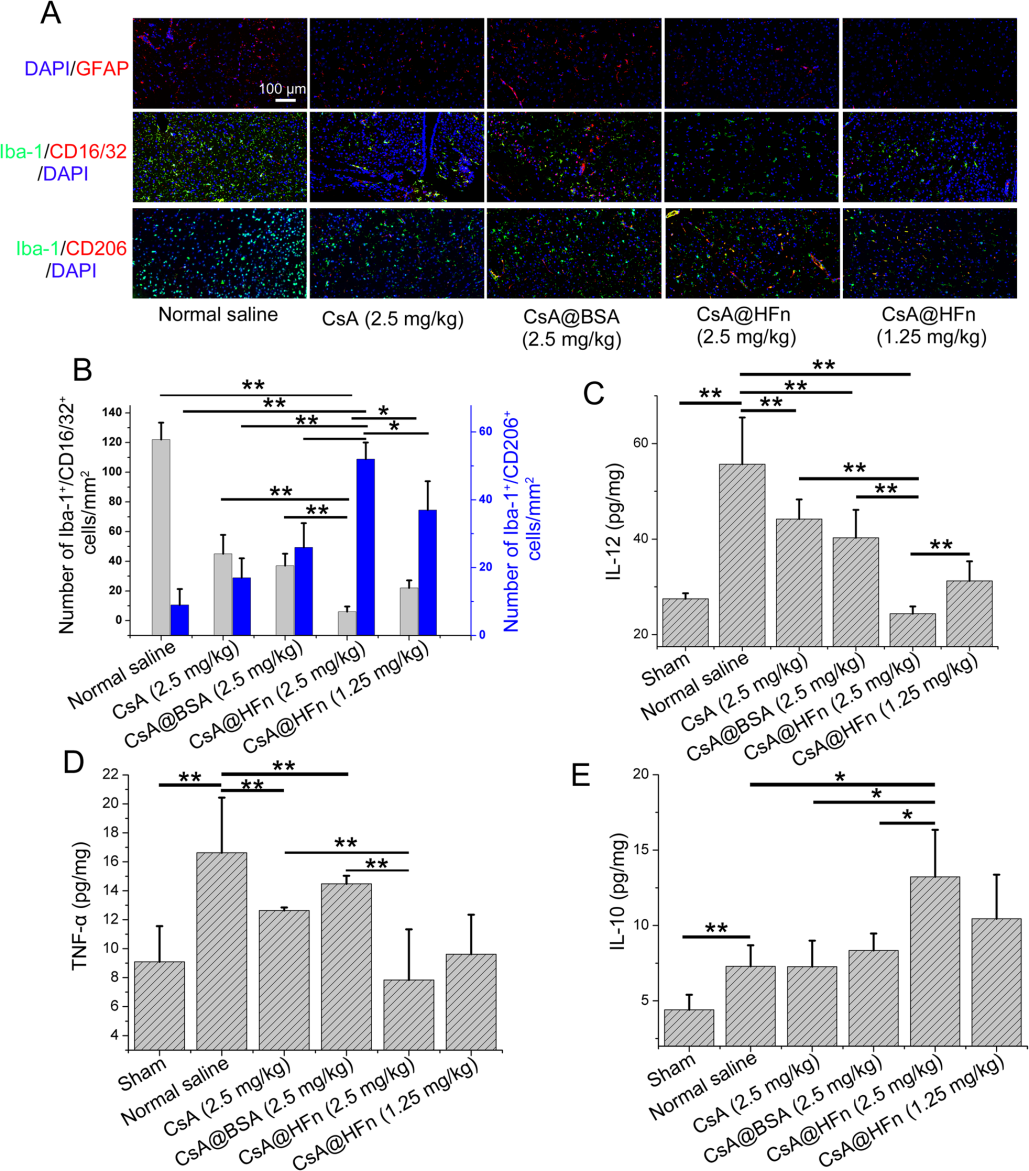
Effects of CsA@HFn on inflammatory microenvironment in cerebral infarction area of MCAO mice. **A** GFAP (astrocyte markers: red color), Iba-1 (microglia markers: green color), CD16/32 (red color) and CD206 (red color) staining of cerebral infarction tissue. **B** Iba-1^+^CD16/32^+^ cells and Iba-1^+^CD206^+^ cells in cerebral infarction tissue. **C–E** The IL-12, TNF-α and IL-10 level in cerebral infarction tissues. (n = 3, x ®±SD, *P < 0.05; **P < 0.01)

Immumofluorescence methods were used to observe the expression of GFAP and Iba-1 in cerebral infarction tissue of MCAO mice. The results showed that the expressions of GFAP and Iba-1 in the normal saline treatment group were much higher than those in the CsA@HFn, CsA and CsA@BSA treatment groups, indicating that cerebral ischemia reperfusion injury caused the recruitment of astrocyte and microglia to cerebral ischemic area in MCAO mice. The number of astrocyte and microglia in cerebral infarction tissue was significantly decreased in the CsA@HFn treatment group (Fig. 6A). In addition, CsA@HFn, CsA and CsA@BSA reduced the number of Iba-1 and CD16/32 co-positive cells and increased the number of Iba-1 and CD206 co-positive cells as compared with the normal saline (Fig. 6A, B), indicating that CsA@HFn, CsA and CsA@BSA reduced the number of M1 type microglia and increased the number of M2 type microglia in cerebral infarction tissue in MCAO mice. As compared with CsA and CsA@BSA, CsA@HFn treated MCAO mice showed more number of M2 type microglia and less number of M1-type microglia in cerebral infarction tissue. The level of IL-12, TNF-α and IL-10 in cerebral infarction tissues was also determined by ELISA method. The results showed that as compared with the sham group, IL-12 and TNF-α level in cerebral infarction tissues in MCAO mice were significantly increased. The IL-12 and TNF-α level were significantly decreased while IL-10 were significantly increased in cerebral infarction tissues in CsA@HFn treated MCAO mice (Fig. 6C–E).

### Effects of CsA@HFn on oxidative stress and BBB integrity in cerebral infarction area in MCAO mice

**Fig. 7:**
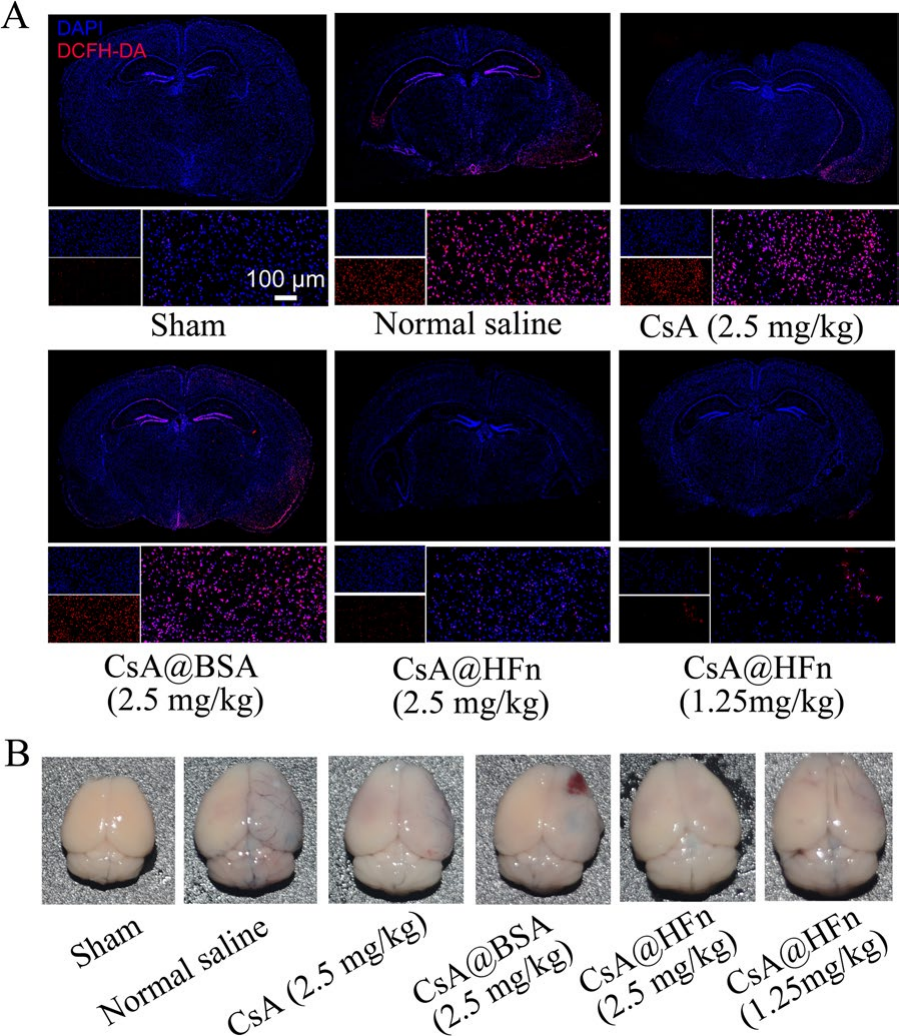
Effects of CsA@HFn on the oxidative stress and BBB integrity in MCAO mice. **A** ROS level detected by DCFH-DA reactive oxygen assay kit in cerebral infarction tissues. **B** Evans blue distributed in brain of MCAO mice

ROS level in cerebral infarction tissues of MCAO mice were detected. Compared with sham group, there were large amount of ROS in cerebral infarction tissues in normal saline treatment group, indicating that cerebral ischemia reperfusion caused oxidative stress in cerebral infarction tissues in MCAO mice. CsA@HFn dose-dependently reduced ROS levels in cerebral infarction tissues in MCAO mice. As compared with CsA and CsA@BSA treated MCAO mice, the ROS level in cerebral infarction tissues was significantly decreased in CsA@HFn treated MCAO mice (Fig. 7A). In addition, the permeability of BBB in MCAO mice was measured by evans blue assay. As shown in Fig. 7B, evans blue could not enter the normal brain tissue. There was more amount of evans blue distributed in the area of cerebral infarction tissue in MCAO model mice, indicating that BBB of MCAO mice was destroyed by cerebral ischemia reperfusion. There was little amount of evans blue distributed in cerebral infarction tissue in CsA@HFn, CsA and CsA@BSA treated MCAO mice. As compared with CsA and CsA@BSA treated MCAO mice, there was less amount of evans blue distributed in cerebral infarction tissue in CsA@HFn treated MCAO mice, indicating that CsA@HFn significantly attenuated the damage of BBB induced by cerebral ischemia reperfusion.

### In vivo preliminary safety evaluation

**Fig. 8:**
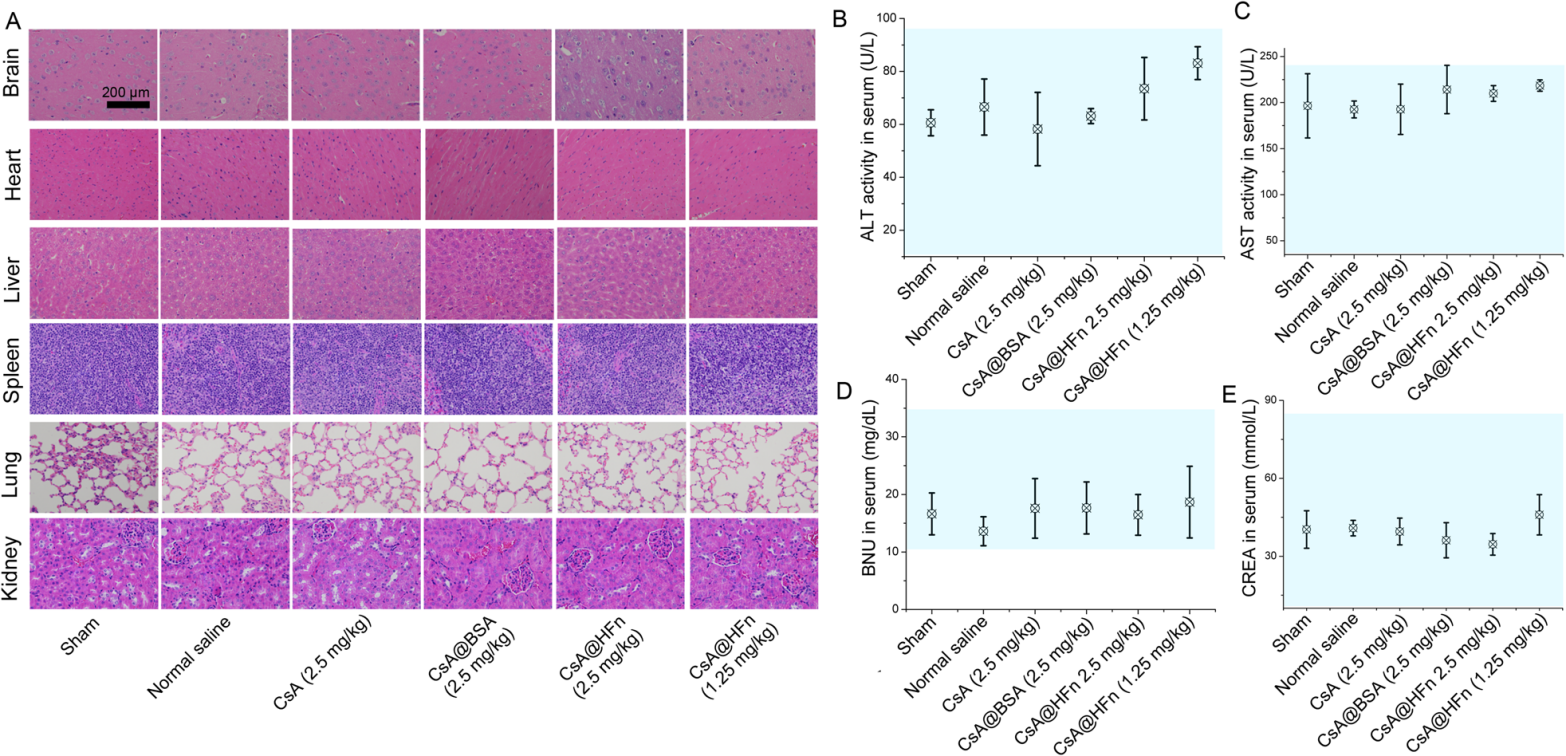
In vivo safety evaluation. **A** *H&E* staining of healthy organs in MCAO mice. **B**, **C** ALT and AST activitity in serum of MCAO mice. **D**, **E** The contents of BNU and CREA in serum of MCAO mice. The blue area indicates the normal ranges. (n = 3, $\stackrel{-}{x}$  ± SD)

*H&E* staining of organ tissue in MCAO mice is showing in Fig. 8A, and there was no obvious morphological change in normal brain, heart, liver, spleen, lung and kidney tissues in drug treated MCAO mice, indicating that CsA, CsA@BSA and CsA@HFn did not cause damage to normal tissues of MCAO mice in the course of treatment. The activities of ALT and AST and the contents of BNU and CREA in serum of MCAO mice in all treatment groups were within the normal range, indicating that CsA@HFn would not cause damage on the liver and kidney functions of MCAO mice in the course of treatment (Fig. 8B–E).

## Discussion

CsA shows great potential in the treatment of cerebral ischemia/reperfusion injury. 127 patients with ischemic stroke were enrolled in a Phase II clinical trial, which was performed to investigate whether intravenous injection of cyclosporine A combined with thrombolytic therapy could reduce infarct size. The results indicated that as compared with placebo, cerebral infarction volume was significantly reduced after intravenous infusion of CsA (2.0 mg/kg), suggesting that CsA played a role in the treatment of ischemic stroke [33]. However, CsA shows poor solubility and non-selective distribution in vivo. It is difficult for CsA to accumulate in the brain after systemic administration. In order to achieve a higher CsA concentration in brain, large dose injection is required. This inevitably causes systemic toxicity [19, 34]. The experimental results indicated that TfR1 was highly expressed on the brain capillary endothelial cells. HFn has a high affinity with TfR1 [35]. Thus, TfR1 can help HFn penetrate into the brain parenchyma by transcytosis [36–38]. Moreover, HFn has a unique self-assembly property [39, 40]. HFn encapsulates CsA through depolymerizing/self-assembling, which greatly reduces the difficulty of clinical transformation of CsA@HFn. Therefore, in this paper, HFn was used to load CsA. The in vitro and in vivo studies indicated that CsA@HFn had a strong ability to penetrate BBB. As compared with CsA@BSA, CsA@HFn displayed much higher transport efficiency across BBB, confirming HFn helped CsA@HFn penetrate BBB. The study also found that the distribution of CsA@HFn in infarcted brain tissue was significantly higher than that in normal brain tissue in MCAO mice, suggesting CsA@HFn was able to target the ischemic brain tissue.

The mPTP plays an important role in cerebral ischemia/reperfusion injury [41]. The over-opening of mPTP produces excessive ROS, leading to the damage of neuron, microglia and blood vessels [42, 43]. Therefore, inhibition of over-opening of mPTP will alleviate cerebral ischemia/reperfusion injury [44]. In vitro experimental results indicated that as compared with free CsA, CsA@HFn significantly reduced the over-opening of mPTP, mitochondrial damage and ROS content in OGD/R injured SH-SY5Y cells. At the same time, the apoptosis of OGD/R injured SH-SY5Y cells was also reduced by CsA@HFn. The above data indicated that CsA@HFn protected SH-SY5Y cells from OGD/R injury through reducing the opening of mPTP, restoring mitochondrial membrane potential and reducing ROS production. In vivo experimental results also showed that CsA@HFn significantly inhibited the ROS outbreak caused by cerebral ischemia/reperfusion, and the inhibitory effect of CsA@HFn on ROS outbreak was stronger than that of CsA and CsA@BSA. This was because more amount of CsA@HFn was distributed in the infarcted brain tissue.

TTC staining, *H&E* staining, nissl staining and silver staining also showed that CsA@HFn significantly reduced the infarct size and neuron injury in cerebral ischemia area. In ischemic stroke, ROS outbreak destroys the tight connections between brain capillary endothelial cells, which lead to the damage of BBB. The in vivo experimental results indicated that CsA@HFn significantly improved the integrity of BBB in MCAO mice, suggesting that CsA@HFn could alleviate neuronal dysfunction, nerve inflammation and neurodegeneration by protecting BBB from damage [45–47].

Microglia is immune effector cells inherent in the central nervous system, and it distributed in gray matter and white matter [44]. Microglia makes up about 10% of the total number of brain cells. Microglia usually remains in a resting state and does not show phagocytosis. They can monitor the state of the microenvironment in the brain parenchyma and remove necrotic neuron in time, maintaining the homeostasis of the central nerve system [48, 49]. Microglia is activated to show its phagocytic function in response to inflammation, infection and trauma [50, 51]. When cerebral ischemia occurs, microglia is rapidly activated and recruited from the peripheral area to the lesion site [52]. At the same time, microglia also undergoes significant phenotypic changes, from resting state to pro-inflammatory M1 type microglia [53]. The M1 type microglia excretes a large number of inflammatory factors such as IL-6, IL-1β, IL-12 and TNF-α, which aggravate cerebral ischemia/reperfusion injury [54]. M2 type microglia has anti-inflammatory effect, it can secrete anti-inflammatory medium and neurotrophic factor such as IL-10, IL-13, TGF-beta, glial cells derived neurotrophic factor (GDNF) and brain-derived neurotrophic factor (BDNF), which promote tissue repair and neuron regeneration in ischemic stroke [55]. Therefore, polarizing M1 type microglia to M2 type microglia will exert therapeutic effects on cerebral ischemia/reperfusion injury. Immunofluorescence staining showed that CsA@HFn significantly reduced the number of astrocytes and microglia in cerebral infarction tissue in MCAO mice as compared with normal saline. Meanwhile, the number of M1 type microglia was significantly reduced and the number of M2 type microglia was markedly increased in cerebral infarction tissue in CsA@HFn treated MCAO mice. ELISA results showed that as compared with normal saline, CsA@HFn significantly decreased the levels of IL-12 and TNF-α and increased the level of IL-10 in cerebral infarction tissue of MCAO mice. This further reduced the production of inflammatory factors in cerebral ischemia area and protected neurons from injury. These results suggested that CsA@HFn not only inhibited the recruitment of astrocyte and microglia to cerebral infarction tissues during cerebral ischemia-reperfusion injury but also polarized the microglia into M2 type microglia, reducing the nerve inflammation and alleviating the injury of neuron in cerebral infarction tissue.

## Conclusions

CsA@HFn specifically delivered CsA to cerebral ischemic region, and subsequently inhibited the over-opening of mPTP in neuron and ROS outburst in cerebral ischemic region. This further improved the integrity of BBB in MCAO mice and reduced the area of cerebral infarction. CsA@HFn significantly inhibited the recruitment of astrocyte and microglia to cerebral infarction tissues while polarized M1 type microglia into M2 type microglia, alleviating the nerve inflammation in ischemic areas and protecting neurons. CsA@HFn has great potential in the treatment of ischemic stroke.

## Data Availability

All data generated or analyzed during this study are included in this published article.

## Abbreviations

ALT: Alanine aminotransferase
AST: Aspartate aminotransferase
ATP: Adenosine triphosphate
BBB: Blood brain barrier
BDNF: Brain-derived neurotrophic factor
BSA: Bovine albumin
CD16/32: Clusters of differentiation 16/32
CD31: Clusters of differentiation 31
CD206: Clusters of differentiation 206
CsA: Cyclosporine A
Cy7: Cyanine 7
DAPI: 4’,6-Diamidino-2-phenylindole
DCFH-DA: 2,7-Dichlorodi -hydrofluorescein diacetate
DMEM: Dulbecco’s modified eagle medium
ELISA: Enzyme linked immunosorbent assay
FAM: 5-Carboxyfluorescein
FBS: Fetal bovine serum
Fn: Ferritin
GDNF: Glial cells derived neurotrophic factor
GFAP: Glial fibrillary acidic protein
HFn: Heavy chain ferritin
*H&E* staining: Hematoxylin-eosin staining
HPLC: High performance liquid chromatography
Iba-1: Ionized calcium binding adaptor molecule 1
IFN-γ: Interferon-γ
IL-10/12: Interleukin-10/12
LFn: Light chain ferritin
LSCM: Laser scanning confocal microscopy
MCAO: Middle cerebral artery occlusion
mPTP: Mitochondrial permeability transition pore
NHS: *N*-Hydroxy succinimide
OGD/R: Oxygen-glucose deprivation/reoxygenation
PBS: Phosphate buffer saline
ROS: Reactive oxygen species
TEM: Transmission electron microscope
Tf: Transferrin
TfR1: Transferrin receptor 1
TNF-α: Tumor necrosis factor-α
TTC: 2,3,5-Triphenyltetrazolium chloride
